# Prevalence of anxiety and depression among married women in Bangladesh: An analysis of nationally representative survey

**DOI:** 10.1371/journal.pmen.0000387

**Published:** 2025-07-22

**Authors:** Md Rabiul Haque, Ahbab Mohammad Fazle Rabbi, Fardin Araf, Md Mostafizur Rahman

**Affiliations:** 1 Department of Population Sciences, University of Dhaka, Dhaka, Bangladesh; 2 Innovision Consulting Private Limited, Dhaka, Bangladesh; 3 Department of Geography and Environmental Science, Begum Rokeya University, Rangpur, Bangladesh; Assiut University, EGYPT

## Abstract

Anxiety and depression are significant factors affecting individuals’ well-being and productivity, yet they often receive inadequate research attention and are not recognized as major public health issues in countries like Bangladesh. This study aims to assess the prevalence and differentials of anxiety and depression among married women in Bangladesh by their different background attributes. Using data from the latest Bangladesh Demographic and Health Survey (BDHS-2022), the mean GAD-7 score was 3.10 (SD = 3.167), and the mean PHQ-9 score was 3.35 (SD = 3.363). This study found that 3.4% of married women suffer from moderate to severe anxiety, while 4.9% experience moderate to severe depression. Moderate to severe anxiety was more prevalent among older women, peaking at 6.5% in the 45–49 age group, women who married before age 15 (5.1%), and those who experienced child mortality (6.2%). Women in the Rangpur division had the highest prevalence of moderate to severe depression (7.6%). These findings underscore the need for targeted interventions addressing both the symptoms and root causes of mental health challenges.

## Introduction

Globally, mental health disorders affected one in eight people in 2019, with anxiety (32%) and depression (29%) being the most prevalent. The prevalence of these two causes of mental health disorders has increased further by 26% and 28% respectively due to COVID-19 [1]. Low- and middle-income countries (LMICs) account for an astounding 82% share of these cases [2,3]. Rapid lifestyle changes in these regions have contributed to the rising prevalence of stress, anxiety, and depression [4,5]. The adversity of these mental health disorders on general health outcomes is critical and cannot be overlooked [6,7]. Mental health disorders are included in the top ten causes of global disease burden and are estimated to be liable for around 11% and 14% of the disease burden in LMICs and globally, respectively [2,8]. The complex, bidirectional relationship between mental and physical health is well-documented and highlights the need for a comprehensive approach to treating mental health issues [9,10]. For example, people with prolonged physical illnesses are more likely to experience mental health disorders, and mental health disorders are the risk factors for chronic physical conditions [10]. While estimates highlight the scope of the issue, it’s important to note that these figures often rely on limited or indirect data sources—especially in LMICs—due to a lack of high-quality, large-scale studies. This data gap means that the true burden of mental health issues may be underestimated or underrepresented in global health discourse [11,12]. As a result, the profound effects of these disorders are sometimes overlooked at the individual level in many LMICs, including Bangladesh, where the widespread belief persists that physical fitness and the absence of illness are the sole indicators of well-being [13].

While anyone can experience anxiety and depression, individuals with a history of abuse, significant loss, or chronic stress are more prone to these conditions. However, compared to men, women are more likely to experience mental health disorders. Women are roughly 50% more likely than men to experience depression [14]. For example, over 10% of expectant and new mothers worldwide suffer from depression [15]. Women in Bangladesh, particularly those who are married at early ages, are socioeconomically disadvantaged due to a range of issues [16]. Women usually move from the parental residence after marriage and sometimes experience difficulties in coping with the husband’s family’s household, particularly for married adolescents. Women who marry at younger ages are less likely to benefit from the potential protective aspects of marriage but are more likely to experience psychological distress [17–19]. Bangladeshi married women in many cases endure physical and mental violence from their intimate partners for multiple reasons [16] although gender-based violence is one of the leading causes of an increased likelihood of anxiety, depression, and even suicidal tendencies among women in many countries [20–22]. Moreover, they have limited voices in the household decision-making process, and choices to move from their typical residences and participate in development activities [23].

Previous studies, mostly with small samples from a specific segment of the population such as students, adolescents, adults, and the elderly have explored the incidence of stress, anxiety, and depression in Bangladesh and other LMICs and identified their determinants [24–28]. For instance, one study focused solely on depression among a limited sample of married adolescent girls aged 15–19 in Bangladesh [29]. Due to the differences in study contexts, target populations, assessment methods, and thresholds for defining mental health disorders, the prevalence of mental health disorders reported in prior Bangladeshi studies varies widely and is often not comparable [11,30,31]. It is also important to note that Bangladesh has not been included in the World Mental Health (WMH) surveys conducted by the World Health Organization. This exclusion may be attributed to limited infrastructure for mental health research, underfunded mental health services, and a historical lack of prioritization of mental health at the national level—factors commonly observed in some low-income countries [32]. Many Bangladeshis continue to face socioeconomic disadvantages like poverty, limited access to healthcare, and poor living conditions, which may contribute to the perception that mental health is not an immediate priority in comparison to physical health needs like infectious diseases, malnutrition, or maternal and child healthcare [12,32]. Although attention to mental health has increased since the adoption of the Mental Health Act in 2018, services remain inadequate, with only 0.05% of the total health budget allocated to mental health [12,32].

However, none of these studies have considered both anxiety and depression among ever-married women aged 15–49 and their differentials using national-level data to examine associated differentials. An in-depth understanding is thus demanded due to the unique circumstances of ever-married women, who are away from their parental families and exposed to different environments with multiple challenges, including early motherhood experience. This study aims to explore the prevalence of anxiety and depression among Bangladeshi ever-married women aged 15–49 years and their differentials by background attributes.

## Data and method

Data for this study come from the most recent Bangladesh Demographic Health Survey (BDHS) 2022. DHS project is responsible for collecting nationally representative data on health and family planning in over 85 low-and-middle-income countries worldwide. DHS surveys are cross-sectional, and Bangladesh DHS surveys are conducted under the authority of the National Institute for Population, Research, and Training (NIPORT) of the Ministry of Health and Welfare. The main objective of the survey is to analyze the health indicators and provide a comprehensive summary of population, maternal, and child health problems.

### Sample design

The BDHS-2022 used a two-stage stratified cluster sample of households. The surveys utilized a list of enumeration areas (EAs) from the 2011 Population and Housing Census of the People’s Republic of Bangladesh, provided by the Bangladesh Bureau of Statistics as the sampling frame. BDHS-2022 used a multistage stratified cluster sampling design. In the first stage, EAs are selected using probability proportional to their size. Following this, a complete household listing is carried out in each selected EA to create the sampling frame for the second-stage household selection. In the second stage, a systematic sample of households from each EA is chosen to ensure statistically reliable estimates of key demographic and health indicators. Only ever-married women in two-thirds of the selected households completed a comprehensive (long) questionnaire that covered all the topics. Considering the response rate, the final sample size became 20029 for the current study. Details of the data extraction process for the current study are plotted below in Table 1.

**Table 1:** Flowchart of the data extraction process for analysis in this study.

Enumeration areas (EA)	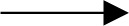	674
Household selected	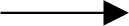	30,330
Total eligible respondents from selected households	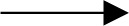	30,078
Eligible women for the long questionnaire (includes anxiety and depression)	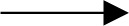	20,220
Study sample (includes anxiety and depression)	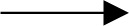	20,029

### Outcome variable

BDHS included two key variables regarding the mental health status of married women (aged 15–49) only for the first time in BDHS-2022 [16]. To evaluate symptoms of anxiety, BDHS utilized the Generalized Anxiety Disorder 7 scale (GAD-7), a set of seven items designed to assess the primary characteristic of anxiety [33]. Each symptom in the GAD-7 is assigned a score of 0, 1, 2, or 3 depending on how frequently the respondent reported experiencing that symptom in the 2 weeks preceding the survey where 0 stands for never; 1 for rarely, 2 means often and 3 represents always. GAD-7 scores range from a minimum of 0 to a maximum of 21. Higher scores are associated with more severe symptoms of anxiety. We categorize it into three categories for summarizing where a GAD-7 score of 0–4 stands for minimal anxiety; 5–9 means mild anxiety and 10 to higher values represent moderate to severe anxiety [34].

To assess symptoms of depression, the module includes nine items from the Patient Health Questionnaire, or PHQ-9 [35]. The questions in the PHQ-9 are based on the Diagnostic and Statistical Manual of Mental Disorders (DSM) criteria for diagnosis of depression (American Psychiatric Association 2013). Both scales focus on symptoms experienced in the 2 weeks preceding the survey. The sum of the scores on each of the nine items in the PHQ-9 forms the overall score. Each symptom in PHQ-9 is assigned a score of 0, 1, 2, or 3 and the interpretation of these values are same as anxiety. PHQ-9 scores range from a minimum of 0 to a maximum of 27. We categorize it into three categories for summarizing where a PHQ-9 score of 0–4 stands for non-minimal or minimal depression; 5–9 means mild depression and 10 to higher values refer to moderate to severe depression [36].

### Explanatory variables

We considered three different levels of explanatory variables for anxiety and depression. Individual-level variables included respondents’ current age, age at first marriage (before 15 years, 15–19 years, and 20 and higher); age at first birth (not teen pregnancy, teen pregnancy); the number of living children (up to 2 and more than 2); experience of child mortality (yes, no); experience of ever having a terminated pregnancy (yes, no); age gap with husband (up to 5 years, 5–10 years, 10–15 years, 15 + years); level of education (primary or lower, secondary or higher); religion (Muslim and non-Muslim); employment status (whether respondent was working or not); mass media exposure status (joint exposure status for newspaper, radio and television); decision making autonomy combining household purchase, freedom of movement (empowered or not); justification of wife-beating (justified or not). We consider only the wealth quintile of the household where the respondent lives (poorer, poor, middle, rich, richer class) as household level. Two explanatory variables have been considered at the community level where the respondents reside: place of residence (urban, rural) and administrative divisions.

### Statistical analysis

Data were analyzed using statistical software Statistical Product and Service Solutions (SPSS). Prevalence of anxiety and depression were measured by mean GAD-7 scores and mean PHQ-9 scores respectively by selected explanatory variables where One-way ANOVA and t-tests were carried out. Additionally, 95% confidence intervals (CIs) were calculated for key prevalence estimates to provide a measure of precision and to assess the reliability of the findings. A chi-square test was conducted to measure the proportional differences in anxiety and depression status across all selected categorical variables. Given the sensitivity of chi-square tests to large sample sizes—where even trivial differences can appear statistically significant—Cramér’s V was also calculated to assess the strength of associations.

### Ethical statement

This study involved human participants; however, the authors did not directly participate in data collection, as this is a secondary data analysis. The survey was conducted by NIPORT and ICF International, with trained staff carrying out the interviews. Verbal consent was obtained from each respondent prior to the interviews, following the informed consent statement. The BDHS data received ethical approval from the ICF Institutional Review Board, in alignment with the ethical standards of the Helsinki Declaration (1964). For this study, we completed the online registration and agreed to all terms and conditions for the use of the BDHS datasets.

## Results

### Distribution of anxiety and depression scores among respondents by selected characteristics

Table 2 presents the mean scores of anxiety (GAD-7) and depression (PHQ-9) among married women in Bangladesh, along with their corresponding standard deviations (SD), 95% confidence intervals (CI), and p-values. The scores are shown across various demographic and socio-economic characteristics of the respondents. Independent sample t-tests were performed for binary explanatory variables, while one-way ANOVA was used for variables with three or more categories to assess group differences. A p-value less than 0.05 was considered statistically significant, indicating that the observed differences in mean scores across groups are unlikely to be due to chance.

**Table 2:** Mean GAD-7 and PHQ-9 scores by different background attributes in Bangladesh (BDHS-2022).

Variables	Mean GAD-7 scores ± SD	95% CI	*p-value	Mean PHQ-9 scores ± SD	95% CI	*p-value
	3.10 ± 3.167	(3.06, 3.14)		3.35 ± 3.363	(3.30, 3.40)	
**Age**						
15-19	2.26 ± 2.752	(2.13, 2.39)	<0.001	2.83 ± 3.199	(2.68, 2.98)	<0.001
20-24	2.47 ± 2.823	(2.37, 2.57)		2.87 ± 3.100	(2.77, 2.98)	
25-29	2.87 ± 3.001	(2.77, 2.97)		3.19 ± 3.221	(3.08, 3.29)	
30-34	3.28 ± 3.214	(3.17, 3.39)		3.42 ± 3.387	(3.31, 3.53)	
35-39	3.47 ± 3.325	(3.36, 3.59)		3.60 ± 3.449	(3.48, 3.72)	
40-44	3.51 ± 3.264	(3.39, 3.64)		3.60 ± 3.449	(3.47, 3.74)	
45-49	3.75 ± 3.445	(3.60, 3.89)		3.92 ± 3.653	(3.77, 4.08)	
**Age at first marriage**						
Before 15 years	3.35 ± 3.230	(3.26, 3.43)	<0.001	3.54 ± 3.460	(3.45, 3.63)	<0.001
15-19	3.01 ± 3.138	(2.95, 3.07)		3.29 ± 3.326	(3.23, 3.35)	
20 and higher	2.96 ± 3.131	(2.85, 3.07)		3.20 ± 3.305	(3.09, 3.32)	
**Had teen pregnancy**						
No	3.07 ± 3.132	(3.00, 3.14)	0.008	3.26 ± 3.310	(3.18, 3.34)	0.001
Yes	3.20 ± 3.196	(3.14, 3.27)		3.43 ± 3.392	(3.37, 3.49)	
**Number of living children**						
Up to 2	2.86 ± 3.046	(2.81, 2.91)	<0.001	3.20 ± 3.294	(3.15, 3.25)	<0.001
More than 2	3.63 ± 3.364	(3.58, 3.68)		3.68 ± 3.491	(3.63, 3.73)	
**Experienced child mortality**						
No	3.03 ± 3.131	(2.98, 3.08)	<0.001	3.29 ± 3.338	(3.24, 3.33)	<0.001
Yes	3.68 ± 3.393	(3.53, 3.83)		3.83 ± 3.523	(3.74, 3.92)	
**Ever had terminated pregnancy**						
No	2.97 ± 3.091	(2.92, 3.02)	<0.001	3.25 ± 3.322	(3.22, 3.28)	<0.001
Yes	3.53 ± 3.375	(3.43, 3.63)		3.68 ± 3.480	(3.58, 3.78)	
**Age gap with husband**						
Up to 5 years	3.01 ± 3.097	(2.93, 3.08)	<0.001	3.28 ± 3.319	(3.20, 3.36)	0.007
5–10 years	2.93 ± 3.009	(2.86, 3.00)		3.20 ± 3.229	(3.13, 3.27)	
10–15 years	3.11 ± 3.207	(3.00, 3.22)		3.38 ± 3.382	(3.26, 3.49)	
15 + years	3.31 ± 3.345	(3.14, 3.48)		3.47 ± 3.415	(3.29, 3.65)	
**Level of education**						
Primary or lower	3.53 ± 3.272	(3.46, 3.60)	<0.001	3.65 ± 3.438	(3.56, 3.74)	<0.001
Secondary or higher	2.82 ± 3.062	(2.79, 2.85)		3.14 ± 3.298	(3.11, 3.18)	
**Religion**						
Muslim	3.14 ± 3.196	(3.12, 3.17)	<0.001	3.38 ± 3.389	(3.33, 3.43)	<0.001
Non-Muslim	2.68 ± 2.841	(2.59, 2.77)		2.98 ± 3.085	(2.89, 3.07)	
**Currently employed**						
No	3.04 ± 3.154	(2.99, 3.09)	<0.001	3.29 ± 3.364	(3.23, 3.35)	0.001
Yes	3.23 ± 3.189	(3.15, 3.31)		3.46 ± 3.359	(3.37, 3.56)	
**Mass media exposure**						
Unexposed	3.21 ± 3.212	(3.14, 3.28)	<0.001	3.33 ± 3.400	(3.26, 3.39)	0.497
Exposed	3.02 ± 3.131	(2.99, 3.05)		3.36 ± 3.336	(3.29, 3.42)	
**Decision-making autonomy**						
Unempowered	2.88 ± 2.936	(2.81, 2.95)	<0.001	3.27 ± 3.164	(3.20, 3.34)	0.785
Empowered	3.11 ± 3.212	(3.05, 3.17)		3.29 ± 3.396	(3.22, 3.36)	
**Wife-beating justification**						
Justified	3.54 ± 3.252	(3.40, 3.68)	<0.001	3.65 ± 3.279	(3.50, 3.80)	<0.001
Not justified	3.02 ± 3.117	(2.97, 3.07)		3.28 ± 3.320	(3.25, 3.31)	
**Wealth quintile**						
Poorest	3.45 ± 3.248	(3.35, 3.56)	<0.001	3.56 ± 3.455	(3.44, 3.67)	<0.001
Poorer	3.26 ± 3.213	(3.16, 3.36)		3.45 ± 3.400	(3.35, 3.56)	
Middle	3.13 ± 3.207	(3.03, 3.23)		3.41 ± 3.444	(3.30, 3.51)	
Richer	3.00 ± 3.155	(2.90, 3.10)		3.28 ± 3.336	(3.18, 3.38)	
Richest	2.70 ± 2.966	(2.61, 2.79)		3.05 ± 3.164	(2.96, 3.15)	
**Place of residence**						
Urban	2.96 ± 3.033	(2.88, 3.05)	<0.001	3.24 ± 3.244	(3.16, 3.32)	0.007
Rural	3.15 ± 3.217	(3.10, 3.20)		3.39 ± 3.409	(3.36, 3.42)	
**Region**						
Barishal	3.25 ± 3.000	(3.08, 3.42)	<0.001	3.43 ± 3.175	(3.25, 3.61)	<0.001
Chattogram	3.30 ± 3.300	(3.19, 3.40)		3.48 ± 3.385	(3.37, 3.59)	
Dhaka	2.79 ± 2.877	(2.71, 2.87)		3.05 ± 3.098	(2.97, 3.14)	
Khulna	3.12 ± 3.413	(2.98, 3.25)		3.51 ± 3.606	(3.36, 3.65)	
Mymensingh	2.76 ± 2.994	(2.61, 2.91)		2.94 ± 3.227	(2.78, 3.10)	
Rajshahi	3.00 ± 2.916	(2.89, 3.11)		3.28 ± 3.198	(3.16, 3.40)	
Rangpur	3.55 ± 3.430	(3.41, 3.69)		3.83 ± 3.726	(3.68, 3.99)	
Sylhet	3.41 ± 3.582	(3.21, 3.62)		3.49 ± 3.677	(3.28, 3.70)	

* The p-value in a t-test or ANOVA indicates whether the difference in mean scores (e.g., PHQ-9 or GAD-7) between two or more groups is statistically significant or likely due to chance. In social sciences, p-values less than 0.05 are usually considered to indicate a statistically significant difference.

The overall mean GAD-7 score was 3.10 ± 3.17. Significant differences in mean anxiety scores were observed across several demographic and socio-economic variables. Younger women (ages 15–19) had the lowest mean anxiety score (2.26 ± 2.75), while women aged 45–49 had the highest mean score (3.75 ± 3.45), with the difference between these age groups being statistically significant (p < 0.001). Women who married before the age of 15 reported higher mean anxiety scores (3.35 ± 3.23) compared to those who married later (3.01 ± 3.14 for ages 15–19 and 2.96 ± 3.13 for 20 and higher). Additionally, women who had more than two children had significantly higher anxiety scores (3.63 ± 3.36) compared to those with fewer children (2.86 ± 3.05) (p < 0.001). Similarly, those who had experienced child mortality reported higher anxiety scores (3.68 ± 3.39) compared to those who had not (3.03 ± 3.13) (p < 0.001). Lower education levels were associated with higher anxiety scores, with women having primary education or lower reporting a mean score of 3.53 ± 3.27 compared to those with secondary or higher education (mean GAD-7 score = 2.82 ± 3.06, p < 0.001).

The overall mean PHQ-9 score was 3.35 ± 3.36. Significant variations in depression scores were also found across several factors. Women aged 45–49 had the highest mean depression score (3.92 ± 3.65), while younger women (ages 15–19) had the lowest score (2.83 ± 3.20), with the difference being statistically significant (p < 0.001). Women who married before age 15 had a higher mean depression score (3.54 ± 3.46) compared to those who married later (3.29 ± 3.33 for ages 15–19 and 3.20 ± 3.31 for 20 and higher, p < 0.001). A significant difference in depression scores was also observed between women who had experienced a terminated pregnancy (3.53 ± 3.38) and those who had not (2.97 ± 3.09, p < 0.001). Women in the poorest wealth quintile reported higher mean depression scores (3.56 ± 3.46) compared to those in the wealthiest quintile (mean = 3.05 ± 3.16, p < 0.001). Furthermore, rural women had slightly higher depression scores (3.39 ± 3.41) compared to urban women (3.24 ± 3.24, p = 0.007).

### Prevalence of anxiety and depression among respondents by selected characteristics

In this section, we examine the prevalence of anxiety and depression, categorized into minimal (non-minimal to minimal), mild, and moderate-to-severe levels, across various demographic and socio-economic attributes. The results are presented in terms of percentage distributions, with *p*-values from chi-square tests indicating statistical significance. A p-value of less than 0.05 suggests a significant association between the background attributes and anxiety/depression levels. This analysis provides further insight into how these factors correlate with mental health outcomes, with the aim of identifying patterns of vulnerability in the population.

Table 3 shows the distribution of anxiety levels (minimal, mild, and moderate-to-severe) across different demographic and socio-economic factors. Younger women, especially those aged 15–19, experience the lowest levels of anxiety, with 82.1% reporting minimal anxiety. In contrast, older women, particularly those aged 45–49, show higher levels of moderate-to-severe anxiety (6.5%), with a statistically significant difference across age groups (*p* < 0.001). Women who married before the age of 15 report higher levels of anxiety, with 5.1% experiencing moderate-to-severe anxiety, a statistically significant finding (*p* < 0.001). Similarly, those with more than two children exhibit higher levels (5.8%) of moderate-to-severe anxiety (*p* < 0.001), as do women who have experienced child mortality (6.2%, *p* < 0.001). Education level also plays a role, with women having primary or lower education showing higher anxiety (5.3% moderate-to-severe anxiety) compared to those with secondary or higher education (3.8%, *p* < 0.001). Employment status, mass media exposure, and decision-making autonomy further impact anxiety levels, with statistically significant associations found in some cases (e.g., underpowered women have higher anxiety levels, *p* < 0.001). However, while the chi-square tests showed statistically significant associations between several background characteristics and anxiety levels, the effect sizes were small in all cases (Cramér’s V < 0.10), indicating weak associations.

**Table 3:** Prevalence of anxiety level by different background characteristics in Bangladesh (BDHS-2022).

Variables	Minimal anxiety (73.5%)	Mild anxiety (22.1%)	Moderate to severe anxiety (3.4%)	Total (n = 20029)	*p-value	**Cramér’s V
**Age**						
15-19	82.1	15.6	2.3	1729	<0.001	
20-24	81.4	15.9	2.7	3290		
25-29	76.7	19.8	3.5	3523		
30-34	71.7	23.4	4.9	3438		0.093
35-39	68.9	25.8	5.3	3344		
40-44	68.1	26.5	5.4	2547		
45-49	65.8	27.7	6.5	2159		
**Age at first marriage**						
Before 15 years	69.9	25.0	5.1	5645	<0.001	
15-19	74.6	21.4	4.0	11313		0.038
20 and higher	75.9	19.7	4.4	3072		
**Had teen pregnancy**						
No	74.7	20.9	4.4	6055	<0.001	0.029
Yes	72.0	23.4	4.5	11879		
**Number of living children**						
Up to 2	76.2	20.0	3.8	13907	<0.001	0.093
More than 2	67.4	26.8	5.8	6121		
**Experienced child mortality**						
No	74.4	21.5	4.2	17854	<0.001	0.058
Yes	66.4	27.4	6.2	2175		
**Ever had terminated pregnancy**						
No	75.1	21.1	3.9	15459	<0.001	0.069
Yes	68.2	25.7	6.1	4570		
**Age gap with husband**						
Up to 5 years	75.3	20.7	4.0	6812	<0.001	
5–10 years	75.1	21.4	3.5	7346		0.027
10–15 years	73.4	22.0	4.6	3445		
15 + years	70.5	23.9	5.6	1457		
**Level of education**						
Primary or lower	68.2	26.5	5.3	7966	<0.001	0.098
Secondary or higher	77.0	19.2	3.8	12062		
**Religion**						
Muslim	73.0	22.5	4.6	18108	<0.001	0..039
Non-Muslim	78.4	18.9	2.7	1921		
**Currently employed**						
No	74.1	21.6	4.3	13616	0.014	0.021
Yes	72.2	23.3	4.6	6412		
**Mass media exposure**						
Unexposed	72.3	23.1	4.6	8433	0.002	0.025
Exposed	74.4	21.4	4.2	11596		
**Decision-making autonomy**						
Unempowered	75.9	20.7	3.4	7721	<0.001	0.032
Empowered	73.6	21.9	4.5	11339		
**Wife-beating justification**						
Justified	66.9	27.3	5.8	2134	<0.001	0.055
Not justified	74.6	21.4	4.0	17546		
**Wealth quintile**						
Poorest	68.9	26.0	5.1	3583	<0.001	
Poorer	71.2	24.7	4.1	4028		
Middle	73.6	21.8	4.6	4136		0.052
Richer	74.8	20.6	4.6	4190		
Richest	78.3	18.1	3.6	4095		
**Place of residence**						
Urban	75.1	20.8	4.1	5700	0.004	0.023
Rural	72.9	22.6	4.5	14328		
**Region**						
Barishal	73.1	22.7	4.2	1199	<0.001	
Chattogram	71.1	24.0	5.0	3749		
Dhaka	78.0	18.6	3.4	5080		
Khulna	73.7	20.5	5.8	2389		0.064
Mymensingh	75.9	20.9	3.2	1527		
Rajshahi	73.5	23.4	3.1	2625		
Rangpur	66.4	27.8	5.7	2290		
Sylhet	72.5	21.7	5.7	1168		

* Note: The p-value in a chi-square test indicates whether the association between two categorical variables is statistically significant or could have occurred by chance. It reflects the exact probability of observing the association between the dependent and independent variables under the assumption that no real association exists in the population. In social science research, p-values less than 0.05 are usually considered statistically significant.

Table 4 presents the prevalence of depression levels—minimal or no depression, mild depression, and moderate-to-severe depression—across various demographic and socio-economic characteristics. Younger women aged 15–19 reported the highest prevalence of minimal or no depression (76.2%), while the lowest was observed among those aged 45–49 (66.2%), with the highest rate of moderate-to-severe depression in that group as well (7.3%, *p* < 0.001). Women who married before the age of 15 showed a higher prevalence of moderate-to-severe depression (5.6%) compared to those who married at age 20 or later (4.4%, *p* < 0.001). Depression was also more prevalent among women with more than two children (5.9%, *p* < 0.001), those who had experienced child mortality (6.8%, *p* < 0.001), or had a history of terminated pregnancy (5.8%, p < 0.001). Educational status showed a clear gradient: women with only primary or lower education had a higher prevalence of moderate-to-severe depression (5.7%) than those with secondary or higher education (4.4%, *p* < 0.001). Religious affiliation (Muslims: 5.0% vs. non-Muslims: 3.7%, *p* < 0.001), employment status (*p* = 0.019), and attitudes toward wife-beating (justified: 5.8% vs. not justified: 4.5%, *p* < 0.001) also showed significant associations with depression levels. Further, poorer mental health outcomes were more prevalent among women from the lowest wealth quintiles, rural areas, and certain regions such as Rangpur and Khulna, where rates of moderate-to-severe depression were notably higher (*p* < 0.001). In line with the findings for anxiety, the chi-square tests for depression revealed statistically significant associations with several background characteristics, but the effect sizes were small in all instances (Cramér’s V < 0.10), suggesting weak practical significance.

**Table 4:** Prevalence of depression level by different background characteristics in Bangladesh (BDHS-2022).

Variables	Non-minimal or minimal depression (71.2%)	Mild depression (23.9%)	Moderate to severe depression (4.9%)	Total (n = 20029)	*p- value	**Cramér’s V
**Age**						
15-19	76.2	20.2	3.6	1729	<0.001	
20-24	76.7	20.0	3.2	3290		
25-29	73.2	22.2	4.6	3523		
30-34	70.5	24.4	5.2	3438		0.063
35-39	68.0	26.6	5.4	3344		
40-44	67.3	27.3	5.4	2547		
45-49	66.2	26.5	7.3	2159		
**Age at first marriage**						
Before 15 years	68.6	25.8	5.6	5645	<0.001	
15-19	72.0	23.2	4.7	11313		0.027
20 and higher	72.8	22.8	4.4	3072		
**Had teen pregnancy**						
No	72.4	23.1	4.5	6055	0.003	0.025
Yes	70.0	24.8	5.2	11879		
**Number of living children**						
Up to 2	72.8	22.7	4.5	13907	<0.001	0.056
More than 2	67.4	26.7	5.9	6121		
**Experienced child mortality**						
No	72.0	23.4	4.7	17854	<0.001	0..050
Yes	64.9	28.3	6.8	2175		
**Ever had terminated pregnancy**						
No	72.5	22.8	4.7	15459	<0.001	0.055
Yes	66.6	27.5	5.8	4570		
**Age gap with husband**						
Up to 5 years	71.8	23.5	4.7	6812	0.003	
5–10 years	73.3	22.5	4.2	7346		0.023
10–15 years	70.0	25.0	5.0	3445		
15 + years	69.4	24.9	5.7	1457		
**Level of education**						
Primary or lower	67.8	26.5	5.7	7966	<0.001	0.062
Secondary or higher	73.5	22.1	4.4	12062		
**Religion**						
Muslim	70.7	24.2	5.0	18108	<0.001	0.031
Non-Muslim	75.4	20.9	3.7	1921		
**Currently employed**						
No	71.8	23.4	4.8	13616	0.019	0.020
Yes	69.9	25.0	5.1	6412		
**Mass media exposure**						
Unexposed	71.7	23.5	4.9	8433	0.447	0.009
Exposed	70.8	24.2	5.0	11596		
**Decision-making autonomy**						
Unempowered	71.3	24.4	4.3	7721	0.17	0.017
Empowered	72.2	22.9	4.9	11339		
**Wife-beating justification**						
Justified	67.0	27.3	5.8	2134	<0.001	0.035
Not justified	71.9	23.5	4.5	17546		
**Wealth quintile**						
Poorest	68.8	25.6	5.6	3583	<0.001	
Poorer	69.8	25.0	5.1	4028		
Middle	70.3	24.4	5.4	4135		0.032
Richer	72.3	23.1	4.6	4189		
Richest	74.4	21.6	4.0	4094		
**Place of residence**						
Urban	72.5	23.1	4.4	5700	0.017	0.020
Rural	70.7	24.2	5.1	14328		
**Region**						
Barishal	70.8	24.5	4.7	1199	<0.001	
Chattogram	69.5	26.3	4.2	3749		
Dhaka	74.6	21.2	4.2	5080		
Khulna	69.9	23.6	6.5	2389		0.060
Mymensingh	74.7	21.5	3.7	1527		
Rajshahi	70.9	25.4	3.7	2625		
Rangpur	65.2	27.2	7.6	2290		
Sylhet	72.6	21.0	6.4	1168		

* Note: The p-value in a chi-square test indicates whether the association between two categorical variables is statistically significant or could have occurred by chance. It reflects the exact probability of observing the association between the dependent and independent variables under the assumption that no real association exists in the population. In social science research, p-values less than 0.05 are usually considered statistically significant. ** Cramér’s V reflects the strength of associations between dependent variable and independent variables.

## Discussion

This study using national-level data of BDHS-2022 attempted to investigate the prevalence’s of anxiety and depression among married women aged 15–49, and their differentials in Bangladesh. According to our study, anxiety and depression are highly prevalent across a range of indicators at the individual, household, and community levels. Our study revealed that anxiety was significantly correlated with women’s age, the experience of pregnancy termination, reduced spousal age gap, lower educational attainment, religion, experiences of physical violence, lower household wealth, region, and higher levels of depression, whereas depression was significantly correlated with experience of child mortality, experience of pregnancy termination, household wealth status, division, and higher levels of anxiety.

Our study reported that 26.5% and 28.8% of ever-married Bangladeshi women suffered from mild to severe anxiety and depressive symptoms respectively. The prevalence of depression is lower than the 53% to 61% reported rates in the prior studies conducted among only adolescents during COVID-19 [19,29]. Variations in the nature and context of the studies as well as the scales employed to measure depression may be linked to the variation in prevalence rates. More specifically, while our study measured depression using PHQ-9 items, the study of Nishat et al. (2023) measured this using DASS-21 of which 7 items were for assessing depression [19]. Hasan & Amin (2024) measured depression with PHQ-9 items only for adolescents [29]. Moreover, data for both of these studies were collected during or immediately after COVID-19 and it is well-documented that COVID-19 leads to an increased rate of depression [37]. Different cultural norms and practices can contribute to the greater prevalence of anxiety and depression in our study. Poor mental health outcomes among married women in Bangladesh were caused by cultural factors such as early marriage, lower subjective happiness, and restricted mobility [38]. Women’s early marriage limits their access to educational attainment and income-generating activities, which in the long run makes them more dependent, hopeless, and depressed [39].

Our study found that the likelihood of anxiety and depression increased with the higher age of married women. In Bangladesh, the average menopausal age is 46.7 years and the greater rate of anxiety and depression among higher-aged groups is likely to be associated with family structure and menopausal transition experience [40–42]. Past studies in line with this study reported that the likelihood of anxiety and depression among middle-aged women in LMICs increased largely during their menopausal transition, as menopause ceases women’s reproductive cycle [41,43]. However, the rates of anxiety and depression by age differences did not show a consistent pattern across the studies [24,29].

Our study revealed that women who have more than two children and who endured pregnancy termination had a greater prevalence of anxiety and depression. Moreover, women’s depression increased significantly with the experience of child mortality. Previous studies conducted in other countries also reported that adverse outcomes of previous pregnancy increased mothers’ anxiety and depression, and infant/child mortality increased mothers’ depressive symptoms [44,45]. Previous research also reported that mothers with more children experienced more depressive symptoms than mothers with fewer children, as rearing children requires substantial social, financial, and healthcare support [46]. Lack of such resources is likely to cause mental stress among mothers. Research indicates that mothers’ anxiety and depression are connected with low socio-economic status [47,48].

According to our research, one of the significant determinants of anxiety and depression among ever-married women was household wealth status. Since socioeconomic status is one of the major social determinants of health and a protective factor of well-being, women from families with lower wealth status were more likely to experience these mental disorders, particularly anxiety. These findings are in line with previous research conducted in Bangladesh and other LMICs, where stress, uncertainty, and a lack of funds to seek healthcare are all intensified by household-level economic instability and lead women to endure anxiety and depression [29,49]. Financial dependence resulting from a lack of educational opportunities and limited involvement in household-level decision-making processes aggravates this vulnerability for many women and creates social exclusion and a sense of helplessness among them [50,51].

Our study also showed that anxiety and depression rates differed significantly by division, which was a novel finding. Compared to other divisions, women in the Rangpur and Chattrogram divisions reported significantly higher levels of anxiety and depression. Due to disaster-induced multiple disadvantages including displacement, household asset damages, and causalities, women in the Rangpur division experienced higher rates of household-level poverty, a lack of social support, restricted access to healthcare and education, and more gender-based violence [52–54]. These disadvantages increase the likelihood of anxiety and depression among Bangladeshi women in disaster-prone areas [53,55]. An estimated 67%, 65%, and 37% of women in Bangladesh who experienced flash floods suffered from severe or extremely severe depression, anxiety, and stress respectively [55]. However, strong religiosity, geographic remoteness, and the concentration of ethnic minorities—where women have limited social mobility and access to education and other well-being services—are likely to be linked to the higher prevalence of anxiety and depression in the Chattrogram Division. Women’s physical and mental health were affected by their unique beliefs attitudes and cultural practices [56]. Indigenous people who lived in Chattrogram’s isolated hill tracts had anxiety and depression levels higher than the clinical threshold [57].

Our study further revealed that women who justify violence by their intimate partner were more likely to suffer from mental health disorders, particularly anxiety. According to previous research, any kind of violence against women has been linked to mental health issues [58,59]. Women who experience violence frequently lose confidence in themselves, which leaves them more susceptible to mental health issues [59,60]. The rates of severe or extremely severe depression, anxiety, and stress increased further among women who experienced family violence during a flood, 89%, 88%, and 58% respectively [55]. Married adolescents in Bangladesh were more likely to experience intimate partner violence due to their limited mobility, early childbearing, short birth intervals, and restrictive social norms [61].

Our study also reported that women with anxiety were more likely to suffer from depression and vice-versa. The relationship between anxiety and depression is complex and vice-versa- one can cause another [62,63]. Past studies also confirmed that anxiety often transformed into depression [64]. People living with anxiety are likely to suffer from depression also [14]. Moreover, the majority of anxiety disorders are primary conditions that significantly raise the risk of developing secondary depression [65,66].

### Strengths and limitations

Unlike most previous studies on the prevalence and determinants of anxiety and depression in Bangladesh, we analyzed data using a nationwide representative sample. Therefore, this study may have suffered less from bias, as participants were not recruited from specialized hospitals or specific regions of the country. We ranked the levels of anxiety and depression on an ordinal scale instead of using a more generalized binary status.

However, our study is not without limitations. We did not adopt a specific framework to identify the correlates of anxiety and depression. We used a modified scale for anxiety and depression, which differs from the original literature, and this may affect the interpretation of the findings. Additionally, we could not examine the association between anxiety and depression by gender and marital status of women due to the data limitations. This study was limited by the data available from the national household survey, which only included mental health data for ever-married women aged 15–49. As such, the results cannot be generalized to unmarried women, men, or older populations. While the focus on ever-married women was driven by the specific research objectives, we acknowledge that comparisons with other demographic groups could provide a more nuanced understanding of mental health issues. Future studies should include a broader range of participants to explore the mental health outcomes of different marital statuses and genders. Finally, although we conducted chi-square tests to explore associations between background characteristics and anxiety/depression, this method is highly sensitive to large sample sizes and may yield statistically significant results even when actual differences are small. To address this, we reported Cramér’s V to assess the strength of associations; however, all effect sizes were small, indicating weak practical significance.

## Conclusions

The determinants of anxiety and depression among married women in Bangladesh are complex and interconnected, influenced by socio-economic conditions, marital dynamics, cultural expectations, and regions. These factors combine to create a challenging environment that affects women’s mental health, underscoring the need for multifaceted interventions. To effectively address these issues, interventions must go beyond merely alleviating symptoms and focus on tackling the root causes of mental health struggles. This includes raising awareness about mental health to reduce stigma, improving access to mental health care services, particularly in rural and underserved areas, and fostering economic empowerment through enhanced job opportunities for women. Additionally, addressing gender-based violence through stronger legal protections and support systems is crucial. By targeting these underlying factors, significant improvements in mental health outcomes for married women in Bangladesh are possible, ultimately enhancing their well-being, economic stability, and overall quality of life.
